# Successful staged hip replacement in septic hip osteoarthritis in osteopetrosis: a case report

**DOI:** 10.1186/1471-2474-13-50

**Published:** 2012-04-02

**Authors:** Giovanni Manzi, Delia Romanò, Laura Moneghini, Carlo L Romanò

**Affiliations:** 1 Dipartimento di Chirurgia Ricostruttiva e delle Infezioni Osteo-articolari, Istituto Ortopedico I.R.C.C.S. Galeazzi, Via Riccardo Galeazzi 4-20166, Milano; 2 Servizio di Anatomia Patologica, Ospedale San Paolo, Via A.Di Rudinì 8-20142, Milano

**Keywords:** Osteopetrosis, Infection, Osteomyelitis, Total hip arthroplasty, Non-union

## Abstract

**Background:**

Osteopetrosis is a rare, inherited, bone disorder, characterized by osteosclerosis, obliteration of the medullary cavity and calcified cartilage. The autosomal dominant form is compatible with a normal life span, although fractures often result from minimal trauma, due to the pathologic nature of bone. Osteomyelitis is common in patients with osteopetrosis because of a reduced resistance to infection, attributed to the lack of marrow vascularity and impairment of white cell function. Only one case of osteomyelitis of the proximal third of the femur has been previously reported, treated with several repeated debridements and finally with femoral head resection. Here we present for the first time a case of a staged implant of a cementless total hip prosthesis for the treatment of a septic hip in femoral neck nonunion in osteopetrosis.

**Case presentation:**

A 36-years-old woman, affected by autosomal dominant osteopetrosis was referred to our department because of a septic hip arthritis associated with femoral neck septic non-union, with draining fistulas. The infection occurred early after a plate osteosynthesis for a closed perthrocanteric fracture of the femur and persisted in spite of osteosynthesis removal, surgical debridement and external fixation. In our hospital the patient underwent accurate debridement, femoral head and greater trochanter resection, preparation of the diaphyseal intramedullary canal and implant of an antibiotic-loaded cement spacer. The spacer was exchanged after one month, due to infection recurrence and four months later, a cementless total hip arthroplasty was implanted, with no clinical and laboratory signs of infection recurrence at two years follow-up.

**Conclusions:**

In case of hip septic arthritis and proximal femur septic non-union, femoral head resection may not be the only option available and staged total hip arthroplasty can be considered.

## Background

Osteopetrosis is a rare inherited bone disorder originally described in 1904 by Albers-Schonberg, a German radiologist [1]; this is a group of sclerosing bone dysplasia due to diminished osteoclast-mediated skeletal resorption. The disorder is characterized by osteosclerosis, obliteration of the medullary cavity and calcified cartilage [2]. Despite the sclerotic radiographic appearance of the thickened cortices and its material hardness, osteopetrotic bone is weak and prone to fracture by minor trauma [3-6]. The sclerosis of bone is, in fact, the result of increased thickness and disorganization, not an increase in mineralization [7-9]. Areas of concentrated stress such as the femoral neck and subtrochanteric areas are especially susceptible [10-12].

Osteopetrosis has been categorised clinically into three primary types: infantile, or "malignant" osteopetrosis, inherited in an autosomal recessive pattern; "intermediate" autosomal recessive osteopetrosis and "benign" autosomal dominant osteopetrosis [2]. The severe infantile forms of osteopetrosis are associated with diminished life expectancy, with most untreated children dying in the first decade as a complication of bone marrow suppression. Orthopaedic surgeons most commonly encounter patients with the benign autosomal-dominant type of osteopetrosis (Albers-Schönberg disease), previously known as adult osteopetrosis or osteopetrosis tarda. In fact autosomal dominant osteopetrosis typically onsets in late childhood or adolescence and is compatible with a normal life span; blood studies show that acid phosphatase, calcitriol, and creatine phosphokinase BB variant levels are elevated, but as many as 40% of patients with the benign form may remain asymptomatic, while most of them first learn of their diagnosis after a fracture [13-15]. Life-threatening symptoms include anemia, pancytopenia, osteomyelitis and sepsis due to poorly developed bone marrow and impaired medullary hematopoiesis, associated with secondary hematopoiesis in liver and spleen that causes hepatosplenomegaly [16-19].

Fractures typically occur in the appendicular skeleton, most commonly in the proximal femur [5,20,21], as well as in the femoral shaft, tibia, and upper extremities [3]. These fractures may result from minimal trauma due to the pathologic nature of bone [3,15]. Fracture-healing abnormalities have been noted both histologically and clinically. Because of dysfunctional remodeling, the callus does not attain haversian organization even by one year postfracture. Broad cement lines persist, creating areas of lowered resistance where microfractures may propagate [11]. Some authors report delayed union and nonunion following fractures [2,15]. In some patients, nonunion of femoral neck fractures may lead to coxa vara [2,3,22,23]. Long-bone deformities are also possible, in particular, lateral bowing of the femur [2]. Coxa vara deformities typically appear during childhood, apparently caused by stress-induced microfractures in the brittle femoral neck [2,22]. Degenerative osteoarthritis also may develop secondary to coxa vara deformity [24,25]. When fractures are encountered, fixation is extremely difficult.

Two of these problems, osteoarthritis and certain periarticular nonunions, may be recalcitrant to other treatment options and may be considered for treatment with total joint arthroplasty. It is recognized that the hard brittle bone, often without a normal medullary canal, makes arthroplasty implantation difficult, may compromise the outcome and lead to more frequent complications [26].

Osteomyelitis is common in patients with osteopetrosis because of a reduced resistance to infection The increased incidence of osteomyelitis has been attributed to the lack of marrow vascularity in osteopetrotic bone and impairment of white cell function [2,4]. The mandible followed by the maxilla, scapula, and extremities is most frequently involved [27-29]. The cause of osteomyelitis of the jaws is usually odontogenic infection and polymicrobial in nature, as opposed to long bone osteomyelitis, in which classically Staphylococcus aureus remains the main responsible organism [2].

To date, only one case of osteomyelitis of the proximal third of the femur has been described and treated successfully with femoral head resection [30]. This is, to our knowledge, the first description of a staged implant of a cementless total hip prosthesis for the treatment of a septic hip in femoral neck nonunion in osteopetrosis

## Case presentation

A 36-years-old woman C.I. was referred to our department in November 2008 because of a septic left hip arthritis and femoral neck non-union, with a draining fistula. Her medical history included the disorder of osteopetrosis (autosomal dominant disorder type II Albers- Schonberg), which was diagnosed at the age of 9. In her lifetime she had suffered a right femoral neck and right tibia diaphyseal fractures.

**Figure 1:**
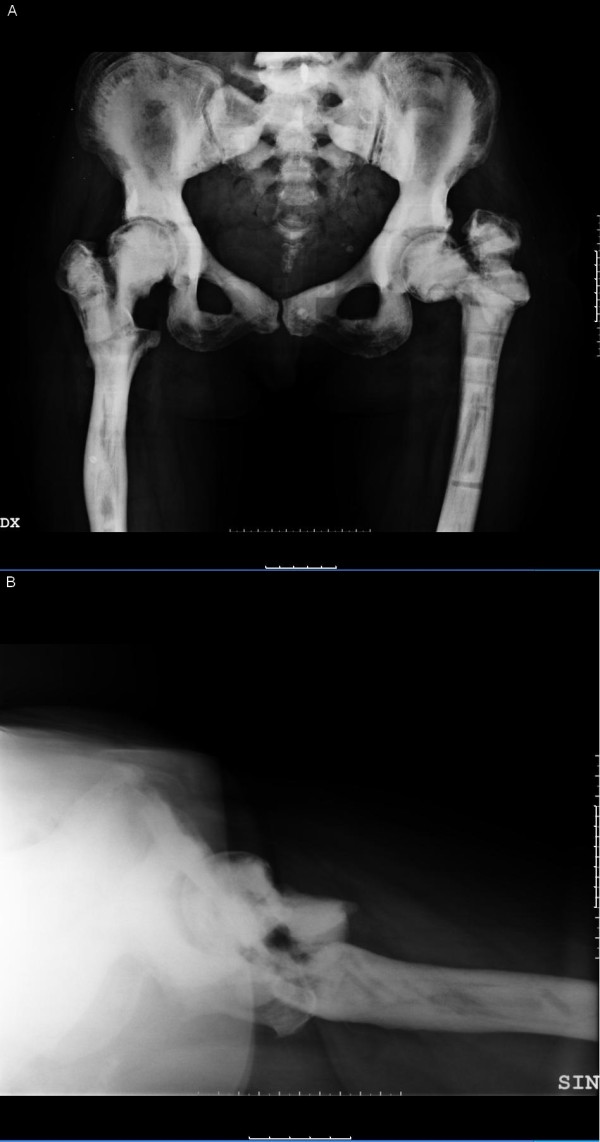
**Antero-posterior (A) and axial (B) x-ray views, pre-operatively, after previous failed plate osteosynthesis and external fixator**. Septic non-union of the femoral neck and septic hip arthritis.

The infection, caused by *Gemella morbillorum*, occurred early after a plate osteosynthesis for a femoral intertrochanteric neck fracture, 12 months before the admission to our department. Following the infection occurrence, she underwent removal of the osteosynthesis, surgical debridement and external fixation. In March 2008 the external fixator was removed, due to infection persistence and draining from the proximal pins. When she came to our observation (November 2008) she presented great functional limitation of the left hip, pain and persistence of the infection, with a draining fistula. X-ray films showed increased bone radiodensity, coxa vara and femoral neck non-union with misalignment of the stumps and the complete absence of an intramedullary canal (Figure 1).

**Figure 2:**
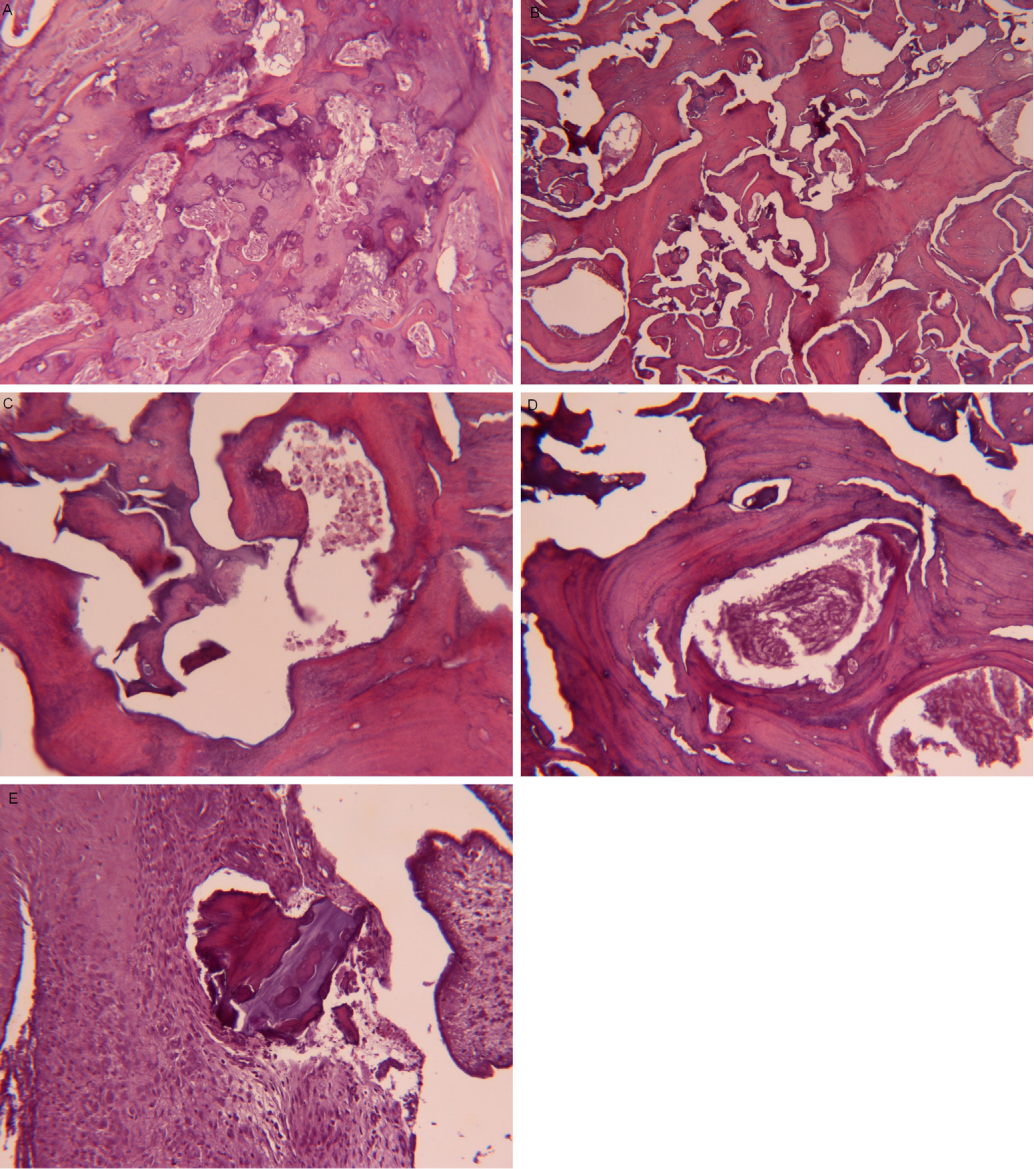
**Trabecular bone made up by remodeling with reactive bone formation (A) and reduction or disappearance of inter-trabecular spaces (B)**. Some residual inter-trabecular space shows micro-aggregation of granulocytes, expression of an acute inflammatory process (C), while other inter-trabecular spaces are filled up with bone matrix and fibrosis (D). At the periphery of the bone, in the sub-synovial tissue, fibrosis with chronic flogosis around bone debris may be observed (E).

We performed a femoral head and greater trochanter resection. During the operation we found a septic pseudoarthrosis of the femoral neck without callus but just fibrotic tissue, that had no mechanic function, and several bone fistulas; it seemed that the infection came out from the femoral head, where they put a cephalic screw. We isolated *Staphylococcus aureus *and *warnerii*. Histological findings are shown in Figure 2.

**Figure 3:**
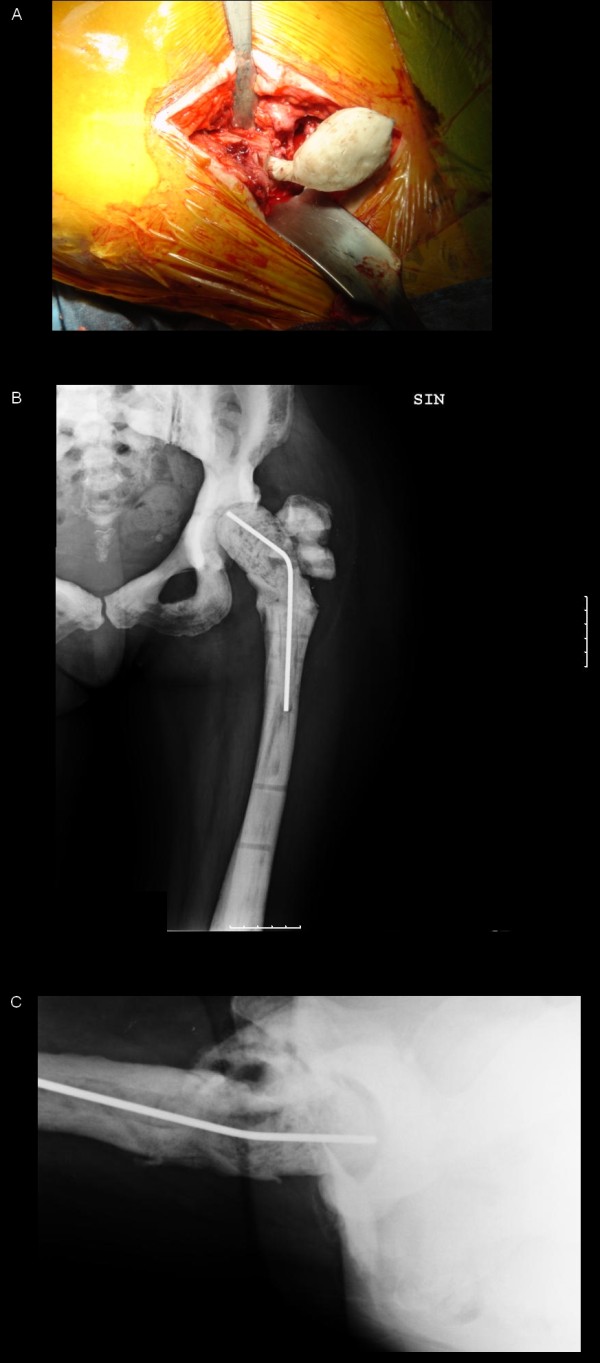
**The custom-made spacer is implanted (A)**. X-rax one month after surgery: antero-posterior (B) and axial view (C).

**Figure 4:**
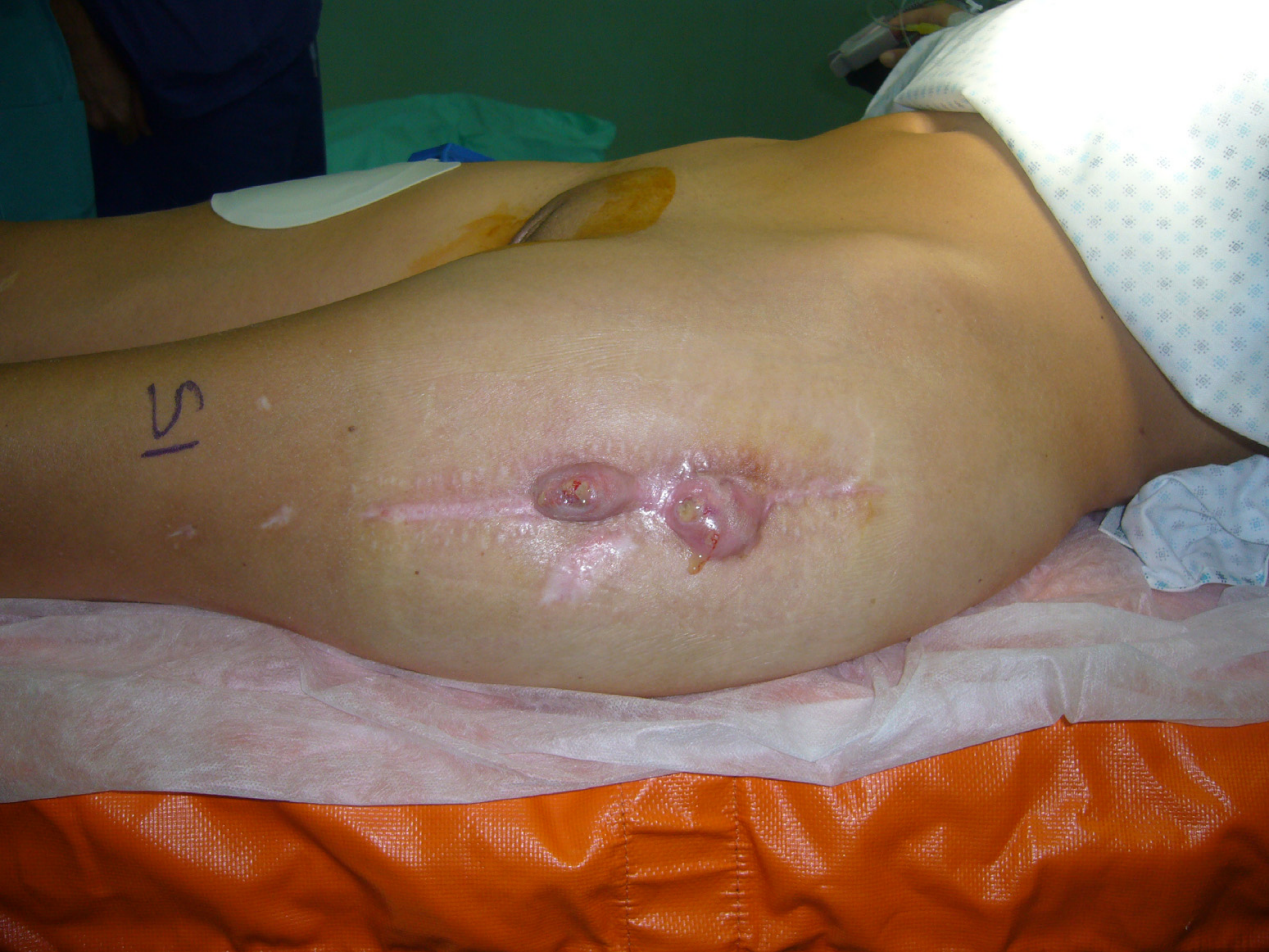
**Clinical picture at the time of infection recurrence, one month after the spacer implant**.

The brittle and hard osteopetrotic bone had sealed off the intramedullary canal completely. Nevertheless, re-canalisation of the diaphyseal intramedullary canal was achieved with a pneumatic burr and under radiological control. After accurate bone preparation, we positioned a preformed antibiotic-loaded (gentamicin and vancomicin) custom-made cement spacer (Figure 3). Systemic antibiotic therapy, vancomicin for two weeks, followed by levofloxacin and rifampicin, was then administered. However, one month later surgery, the patient presented at the Emergency Department of our Institute because of hyperpyrexia and two draining fistulas at the operated hip (Figure 4). A second debridement and soft tissue curettage was then performed, with the removal of the old spacer and the exchange with a new one, loaded with gentamicin and vancomycin. Microbiological cultures of surgical samples grew *Enterococcus spp*.

**Figure 5:**
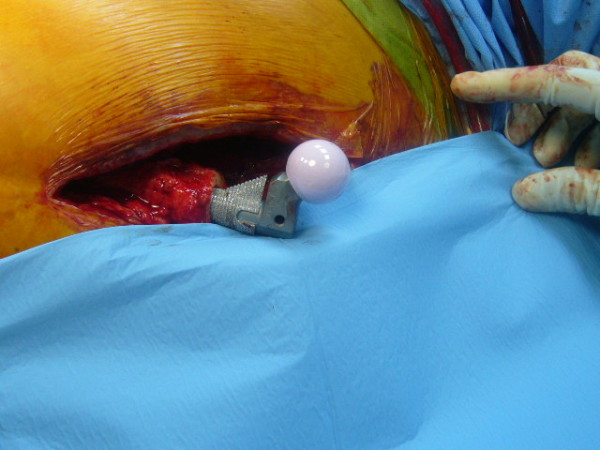
**Intra-operative picture, at the time of cementless hip prosthesis implant**.

Four months later, in the absence of clinical signs of infection and normal C-reactive protein serum levels, we removed the spacer and implanted a cementless modular hip revision prosthesis (S-ROM, Pinnacle, Johnson & Johnson-DePuy Inc) (Figure 5). All the intra-operative microbiological samples were negative.

**Figure 6:**
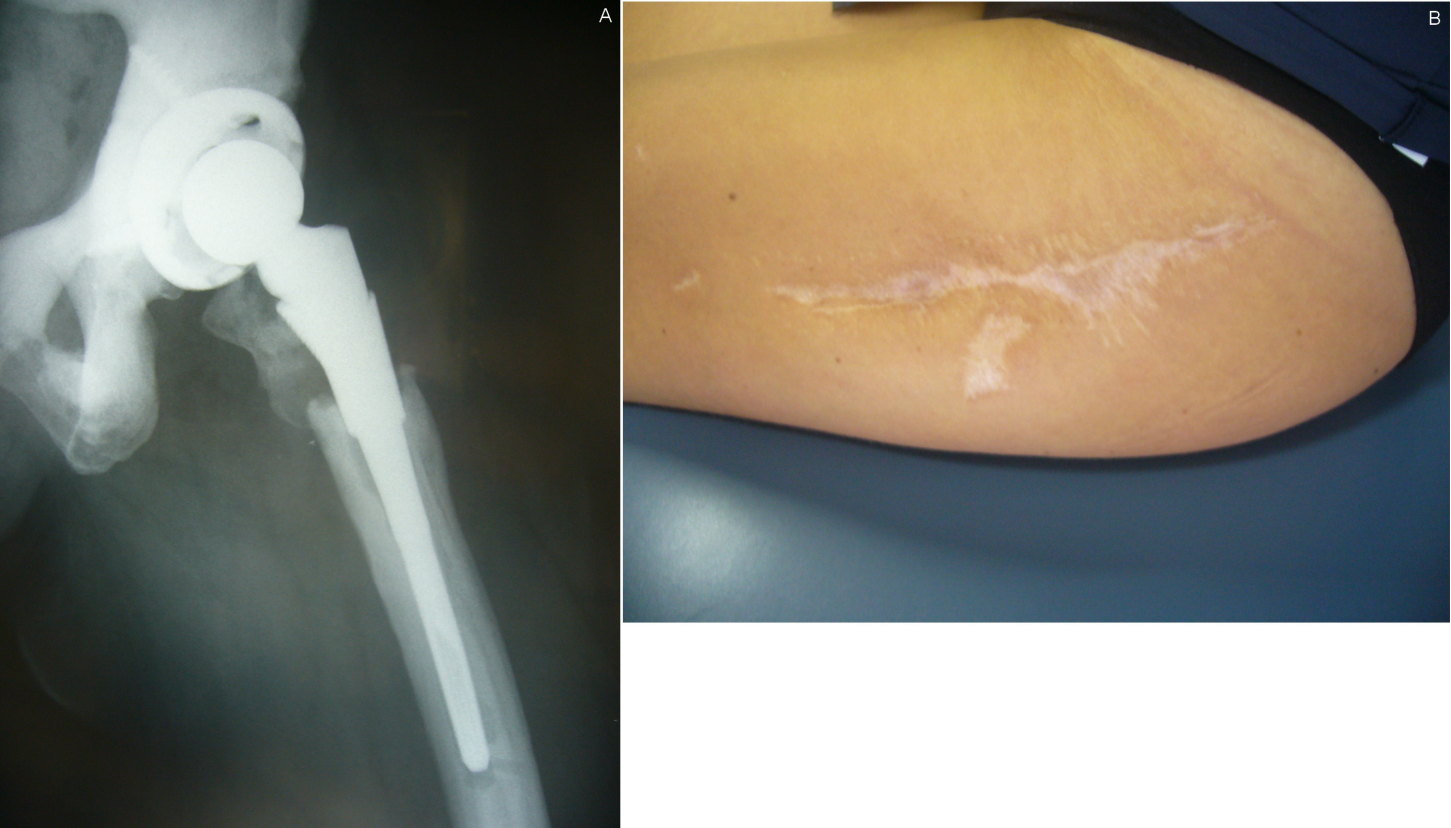
**X-ray (A) and clinical picture two years after surgery**.

At 2-year follow-up, there are no clinical signs of infection recurrence while laboratory tests remain in the normal range value and the prosthetic components do not show any sign of osteolysis at the radiographic examination (Figure 6). The patient, although pain-free, still requires one crutch, due to a persistent weakness of the abductor muscles and a positive Trendelenburg sign.

## Discussion

At present, no effective medical treatment for osteopetrosis exists. Treatment is largely supportive and is aimed at providing multidisciplinary surveillance and symptomatic management of complications. Fractures and arthritis are common and require treatment by an experienced orthopaedic surgeon due to the brittleness of the bone, and the relatively frequent occurrence of secondary complications such as delayed union or non-union of fractures and osteomyelitis [19].

Degenerative changes often occur after the age of 40 [2,5,24,31-33] in the absence of deformity. Articular cartilage is not affected by the disorder, and it has therefore been suggested that the degeneration occurs because of the hard unyielding subchondral bone [24,32].

Many intraoperative and postoperative difficulties have been encountered in patients with osteopetrosis. The poor quality of bone complicates surgery because of its resistance to drilling and tendency to fracture with minimal trauma, resulting in increased surgical time.

In case of osteomyelitis, surgical débridement and "en bloc" resection are necessary [23,30]. Aggressive treatment is indispensable because once chronic foci are formed, they are extremely difficult to eradicate [21].

Another challenge in the management of osteopetrotic patients is arthroplasty. The hardness of osteopetrotic bone and obliteration of the medullary cavity precludes the use of hand reamers [34]. The surgeon must recreate a medullary canal with a highspeed burr or power drill, a difficult process with many technical problems [26,32]. So it could be useful a small femoral stem. There is a high risk of iatrogenic fracture during implant placement, so it could be useful fluoroscopic guidance. Of course there is an increased risk of infection intraoperatively. Even with these modifications, the average surgical time in primary total hip arthroplasty is 5 hours [26].

Despite difficulties in femoral canalization in these patients, total joint arthroplasty has proved to be effective in treating osteoarthritis and periarticular fractures. Three total knee and 15 total hip arthroplasties have been reported in the English-language literature to date [23,26,32,34]. Of these 15 cases, only 2 had postoperative complications (although 1 was lost to follow-up)[26]. Cementless total hip arthroplasty also has been attempted. Cementless acetabular component fixation has been used because it does not require cancellous bone, which is sparse in osteopetrosis patients, for bone-cement fixation [34].

Rolauffs et al. reported the only case available in the English literature, that, similarly to ours, did presented a chronic osteomyelitis following internal fixation of a proximal femoral fracture in a patient affected by osteopetrosis; in that case, the authors report that four debridements were required to manage the recurrent infection. Weakening of the bone finally occurred, and the patient suffered a femoral neck fracture, that obliged to a resection arthroplasty. In the light of our result, we cannot agree with the statement of these authors that "treatment options such as arthroplasty of the osteomyelitic bone" should be ruled out in these patients, while we may suggest to consider a staged hip cementless implant.

## Conclusions

Total joint arthroplasty may be considered as a last option to treat osteopetrosis associated with osteoarthritis. However, many intraoperative and postoperative challenges need to be overcome when performing arthroplasty in patients with osteopetrosis. The greatest challenge in all these surgical procedures is the creation of an intramedullary canal in osteopetrotic bone without a semblance of an intramedullary canal. This is the first report, to our knowledge, that shows staged total hip replacement as a successful option for the treatment of chronic osteomyelitis of the proximal third of the femur and septic hip arthritis.

### Consent

Written informed consent was obtained from the patient for publication of this case report and any accompanying images. A copy of the written consent is available for review by the Editor-in-Chief of this journal.

## Competing interests

The authors declare that they have no competing interests.

## Authors' contributions

GM wrote the draft of the manuscript and participated in the follow-up examination of the patient and clinical material. DR and NL participated in the surgical and medical treatment and followed up the patient. They also have been involved in drafting the manuscript or revising it critically. CLR performed the surgery, coordinated and helped to draft and finalize the manuscript. All authors read and approved the final manuscript.

## Authors' information

CLR is the Director of the Centro di Chirurgia delle Infezioni Osteo-articolari of the research orthopaedic institute Galeazzi in Milano, Italy. Past -president of the Italian Studygroup on Osteoarticular Infecitons, he actually serves as President of the European Bone and Joint Infection Society.

## Pre-publication history

The pre-publication history for this paper can be accessed here:

http://www.biomedcentral.com/1471-2474/13/50/prepub

## References

[B1] Albers-Schonberg H Rottgenbilder einer selten Knochenerkrankung Munchen Med Wchnschr 1904 51 365

[B2] Shapiro F Osteopetrosis: current clinical considerations Clin Orthop 1993 294 34 44 8358940

[B3] Armstrong DG Newfield JT Gillespie R Orthopedic management of osteopetrosis: results of a survey and review of the literature J Pediatr Orthop 1999 19 122 132 9890301

[B4] Chhabra A Westerlund LE Kline AJ McLaughlin R Management of proximal femoral shaft fractures in osteopetrosis: a case series using internal fixation Orthopedics 2005 28 587 592 16138472 10.3928/0147-7447-20050601-15

[B5] Milgram JW Jasty M Osteopetrosis: a morphological study of twenty-one cases J Bone Joint Surg Am 1982 64 912 929 7085720

[B6] Del Fattore ACA Teti A Genetics, pathogenesis and complications of osteopetrosis Bone 2008 42 19 29 10.1016/j.bone.2007.08.029 17936098

[B7] Manolagas SC Birth and death of bone cells: basic regulatory mechanisms and implications for the pathogenesis and treatment of osteoporosis Endocr Rev 2000 21 115 137 10.1210/er.21.2.115 10782361

[B8] Walker DG The classic: osteopetrosis cured by temporary parabiosis Clin Orthop Relat Res 1982 162 2 3 7039911

[B9] Kovanlikaya A Loro ML Gilsanz V Pathogenesis of osteosclerosis in autosomal dominant osteopetrosis AJR Am J Roentgenol 1997 168 929 932 9124142 10.2214/ajr.168.4.9124142

[B10] Casden AM Jaffe FF Kastenbaum DM Bonar SF Osteoarthritis associated with osteopetrosis treated by total knee arthroplasty: report of a case Clin Orthop Relat Res 1989 247 202 207 2791389

[B11] de Palma L Tulli A Maccauro G Sabetta SP del Torto M Fracture callus in osteopetrosis Clin Orthop Relat Res 1994 308 85 89 7955707

[B12] el-Tawil T Stoker DJ Benign osteopetrosis: a review of 42 cases showing two different patterns Skeletal Radiol 1993 22 587 593 8291011 10.1007/BF00197140

[B13] Bhargava A Vagela M Lennox CM "Challenges in the management of fractures in osteopetrosis" Review of literature and technical tips learned from long-term management of seven patients Injury 2009 40 11 1167 1171 10.1016/j.injury.2009.02.009 19576583

[B14] Marks SC Pathogenesis of osteopetrosis in the rat: reduced bone resorption due to reduced osteoclast function Am J Anat 1973 138 165 178 10.1002/aja.1001380204 4747736

[B15] Bollerslev J Mosekilde L Autosomal dominant osteopetrosis Clin Orthop Relat Res 1993 294 45 51 8358946

[B16] Van Hul W Vanhoenacker F Balemans W Janssens K De Schepper AM Molecular and radiological diagnosis of sclerosing bone dysplasias Eur J Radiol 2001 40 198 207 10.1016/S0720-048X(01)00400-4 11731208

[B17] Whyte MP Royce PM, Steinman B Osteopetrosis Connective tissue and its heritable disorders: medical, genetic, and molecular aspects. Ed2 2002 New York: Wiley-Liss, Inc 753 770

[B18] Van Wesenbeeck L Van Hul W Lessons from osteopetrotic mutations in animals: impact on our current understanding of osteoclast biology Crit Rev Eukaryot Gene Expr 2005 15 133 162 10.1615/CritRevEukaryotGeneExpr.v15.i2.40 16022633

[B19] Key LL Rodriguiz RM Willi SM Long-term treatment of osteopetrosis with recombinant human interferon gamma N Engl J Med 1995 332 1594 1599 10.1056/NEJM199506153322402 7753137

[B20] Landa J Margolis N Di Cesare P Orthopaedic management of the patient with osteopetrosis J Am Acad Orthop Surg 2007 15 654 662 17989416 10.5435/00124635-200711000-00004

[B21] Gupta R Gupta N Femoral fractures in osteopetrosis: case reports J Trauma 2001 51 997 999 10.1097/00005373-200111000-00027 11706352

[B22] Gwynne-Jones DP Hodgson BF Hung NA Bilateral, uncemented total hip arthroplasty in osteopetrosis J Bone Joint Surg Br 2004 86 276 278 10.1302/0301-620X.86B2.14354 15046446

[B23] Ashby ME Total hip arthroplasty in osteopetrosis: a report of two cases Clin Orthop Relat Res 1992 276 214 221 1537156

[B24] Cameron HU Dewar FP Degenerative osteoarthritis associated with osteopetrosis Clin Orthop Relat Res 1977 127 148 149 912970

[B25] Girard J Vendittoli PA Lavigne M Roy AG Resurfacing arthroplasty of the hip in osteopetrosis J Bone Joint Surg Br 2006 88 818 821 16720780 10.1302/0301-620X.88B6.17419

[B26] Strickland JP Berry DJ Total joint arthroplasty in patients with osteopetrosis: a report of 5 cases and review of the literature J Arthroplasty 2005 20 815 820 10.1016/j.arth.2004.11.015 16139724

[B27] Tabrizi R Arabi AM Arabion HR Gholami M Jaw osteomyelitis as a complication in osteopetrosis J Craniofac Surg 2010 21 136Y141 20072016 10.1097/SCS.0b013e3181c46df2

[B28] Hwang JM Kim IO Wang KC Complete visual recovery in osteopetrosis by early optic nerve decompression Pediatr Neurosurg 2000 33 328 10.1159/000055980 11182645

[B29] Kocher M Kasser J Osteopetrosis Am J Orthop 2003 32 222 12772872

[B30] Rolauffs B Bernhardt TM von Eiff C Hart ML Bettin D Osteopetrosis, femoral fracture, and chronic osteomyelitis caused by Staphylococcus aureus small colony variants (SCV) treated by Girdlestone resection: 6-year follow-up Arch Orthop Trauma Surg 2002 122 547 550 12483342 10.1007/s00402-002-0435-2

[B31] Janecki CJ Nelson CL Osteoarthritis associated with osteopetrosis treated by total hip replacement arthroplasty: report of a case Cleve Clinic Q 1971 38 169 177 10.3949/ccjm.38.4.169 5166044

[B32] Casden AM Jaffe FF Kastenbaum DM Bonar SF Osteoarthritis associated with osteopetrosis treated by total knee arthroplasty: report of a case Clin Orthop 1989 247 202 207 2791389

[B33] Siegal A Delling G Total hip joint endoprosthesis in osteoporosis Chirug 1992 63 984 987 1458995

[B34] Matsuno T Katayama N Osteopetrosis and total hip arthroplasty: report of two cases Int Orthop 1997 21 409 411 9498153 10.1007/s002640050196 PMC3619559

